# Lysophosphatidylserine Nanoparticles Induce Oral Tolerance Independent of TIM-4 and Canonical PS Processing

**DOI:** 10.64898/2026.09.24.754158

**Published:** 2026-09-29

**Authors:** Edwin J. Ovalle, Vincent Chak, Elizabeth A. Wohlfert, Sathy Balu-Iyer, Jason G. Kay

## Abstract

Protein replacement therapy is highly effective for treating many protein-deficiency disorders, but its long-term clinical benefit is often limited by anti-drug antibody responses that neutralize therapeutic proteins, compromising safety and efficacy. To address these unwanted immune responses, a lipid nanoparticle–based immunotherapy platform has been developed to induce antigen-specific tolerance. Mice treated with antigen associated with either double-chain phosphatidylserine (PS) or lysophosphatidylserine (LysoPS), a single-acyl-chain phospholipid, showed reduced antibody titers following subcutaneous pre-exposure. However, only LysoPS nanoparticles induced tolerance after oral administration, suggesting structural differences between PS and LysoPS can impact tolerogenic potential, depending on the site of delivery. As an apoptotic “eat-me” signal, PS is recognized by phagocytic receptors such as TIM-4 and is associated with LC3-associated phagocytosis. These nanoparticles have been hypothesized to promote “apoptotic mimicry” to regulate immunity. However, the contribution of TIM-4 to oral tolerance remains unclear. Here, we investigated the role of TIM-4 in LysoPS-mediated oral tolerance using a knockout mouse model. Our data showed that TIM-4 ^-/-^ mice induced tolerance to ovalbumin encapsulated in LysoPS comparable to that of wild-type mice. We also found that LysoPS nanoparticles did not follow the same surface engagement and intracellular trafficking pathways as PS nanoparticles. These results reveal that, despite sharing the same headgroup, differences in acyl chain number critically shape nanoparticle–immune cell interactions and provide a mechanistic framework that may explain the unique ability of LysoPS nanoparticles to induce oral tolerance.

## Full Text Availability

The license terms selected by the author(s) for this preprint version do not permit archiving in PMC. The full text is available from the preprint server.

